# The Adverse Effects of Alcohol on Vitamin A Metabolism

**DOI:** 10.3390/nu4050356

**Published:** 2012-05-07

**Authors:** Robin D. Clugston, William S. Blaner

**Affiliations:** Department of Medicine and Institute of Human Nutrition, College of Physicians and Surgeons, Columbia University, New York, NY 10032, USA; Email: wsb2@columbia.edu

**Keywords:** ethanol, retinol, retinyl ester, retinoic acid, hepatocyte, hepatic stellate cell, cytochrome P450, alcohol dehydrogenase, aldehyde dehydrogenase

## Abstract

The objective of this review is to explore the relationship between alcohol and the metabolism of the essential micronutrient, vitamin A; as well as the impact this interaction has on alcohol-induced disease in adults. Depleted hepatic vitamin A content has been reported in human alcoholics, an observation that has been confirmed in animal models of chronic alcohol consumption. Indeed, alcohol consumption has been associated with declines in hepatic levels of retinol (vitamin A), as well as retinyl ester and retinoic acid; collectively referred to as retinoids. Through the use of animal models, the complex interplay between alcohol metabolism and vitamin A homeostasis has been studied; the reviewed research supports the notion that chronic alcohol consumption precipitates a decline in hepatic retinoid levels through increased breakdown, as well as increased export to extra-hepatic tissues. While the precise biochemical mechanisms governing alcohol’s effect remain to be elucidated, its profound effect on hepatic retinoid status is irrefutable. In addition to a review of the literature related to studies on tissue retinoid levels and the metabolic interactions between alcohol and retinoids, the significance of altered hepatic retinoid metabolism in the context of alcoholic liver disease is also considered.

## 1. Introduction

**Figure 1:**
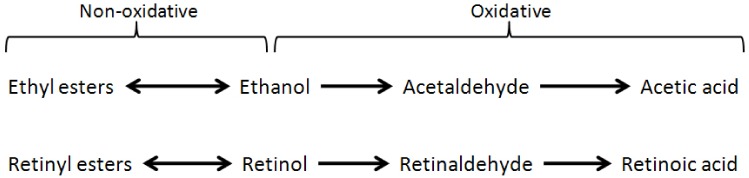
Biochemical parallels in the metabolism of ethanol and retinol. The conversion of ethanol to acetaldehyde is mediated by different processes within the cell; this reaction can be catalyzed by alcohol dehydrogenase, CYP2E1, and to a lesser extent, catalase. The conversion of the primary alcohol, retinol, to retinaldehyde can also be catalyzed by alcohol dehydrogenase subtypes, as well as specific retinol dehydrogenases. In both cases, the subsequent oxidation of the aldehyde to a carboxylic acid is mediated by aldehyde dehydrogenases. Within the liver, the synthesis of retinyl ester is catalyzed by lecithin: retinol acyltransferase (LRAT); the molecular identity of enzymes which synthesize fatty acid ethyl esters are currently unknown.

The primary pathway for ethanol metabolism in the human body is its oxidative metabolism from ethanol to acetaldehyde, and then to acetic acid. Furthermore, ethanol can also undergo so called non-oxidative metabolism, which includes the esterification of ethanol into fatty acid ethyl esters, as well as its conversion into phosphatidylethanol [1,2]. Similarly, the metabolism of dietary vitamin A follows a parallel pathway, including the oxidative metabolism of retinol to retinaldehyde, then to the canonically active form of vitamin A, retinoic acid (collectively referred to as retinoids; Figure 1) [3]. Given these biochemical parallels, it is perhaps not surprising that ethanol has been shown to negatively affect retinoid homeostasis following both acute and chronic ethanol exposure. Indeed, as discussed below, enzymes which are involved in ethanol metabolism are also thought to affect retinoid homeostasis, such as alcohol dehydrogenase and cytochrome P450 2E1 (CYP2E1). The scope of this review includes alcohol’s effect on retinoid metabolism and homeostasis in adult tissues, particularly the liver; however, it should be noted that ethanol has also been proposed to disrupt retinoid signaling during development which has been associated with fetal alcohol syndrome. This subject will not be covered in the current review and the reader is directed toward the existing review literature on fetal alcohol syndrome and retinoids [4,5]. 

## 2. Vitamin A Deficiency and Alcoholism

It has long been recognized that alcoholics are generally malnourished and can suffer from vitamin A deficiency (VAD) [6,7]. A common consequence of alcoholism is loss of night vision (nyctalopia), a condition that is associated with VAD [7,8,9,10]. While this manifestation of VAD is relatively mild, the ophthalmic symptoms of continued VAD include xerophthalmia and can ultimately lead to blindness. Though this is a rare occurrence in alcoholics, there are case reports of xerophthalmia in this patient group [11]. In addition to clinical manifestations of VAD, several studies have reported biochemical measures of vitamin A status in alcoholics. Decreased plasma retinol levels and plasma retinol binding protein (RBP) concentrations have been reported in alcoholics by several groups [10,12,13]; however, it was the landmark 1982 study by Leo and Lieber that established the profound effect that alcohol can have on hepatic vitamin A status [14]. In their manuscript, these authors reported the hepatic vitamin A levels of control subjects and compared them to patients with advancing stages of alcoholic liver disease (fatty liver, hepatitis, cirrhosis), finding a significant decrease in hepatic retinoid content which correlated with disease severity. These authors also confirmed the previously observed decrease in plasma retinol and RBP levels in alcoholics, though only in those patients with more advanced stages of disease (hepatitis and cirrhosis). This latter observation was significant because it emphasized that alcoholics with plasma retinol levels in the normal range may still have significant depletion of hepatic retinoid stores. The observed decrease in hepatic retinoid content of alcoholics has been independently confirmed; it has also subsequently been shown that retinoid levels are decreased in both hepatocytes and hepatic stellate cells (HSC). Furthermore, separate quantification of hepatic retinol and retinyl ester concentrations found that both retinoid species were decreased in the livers of alcoholics [15,16,17].

While it is apparent that alcoholics often have a generalized nutrient deficiency, research in alcohol-fed baboons and rodents established that alcohol was able to deplete hepatic retinoid stores independent of dietary vitamin A intake and absorption [6,7,18]. Thus, it is clear that chronic consumption of alcohol in humans causes a progressive depletion of hepatic retinoid stores, and that alcoholics are prone to developing clinical symptoms of VAD. What is less clear, however, is the effect that altered retinoid homeostasis has on progression of alcohol-induced disease, a topic which is discussed below in Section 5.

## 3. Effect of Alcohol Consumption on Tissue Retinoid Levels

Following the observed effects of alcohol on retinoid homeostasis in humans, numerous studies in animal models have been undertaken to identify the mechanisms through which alcohol affects retinoid metabolism, as well as the impact this may have on the development of alcohol-induced disease. As mentioned above, depleted hepatic retinoid content observed in humans has also been observed in baboons and rodents chronically fed alcohol. Sato and Lieber fed a cohort of baboons alcohol and compared them to control animals, observing decreased hepatic retinoid content after 4 months of alcohol consumption, which was further depleted in a time-dependent manner with up to 2 years of alcohol consumption [18]. While this research carried out in primates is perhaps most applicable to human health and disease, the majority of research studying the interactions between alcohol and vitamin A has been performed in rodents (summarized in Table 1). Nevertheless, a consistent pattern emerges: chronic alcohol consumption is associated with a significant decrease in hepatic retinol and retinyl ester content. Interestingly, many animal studies have failed to recapitulate the decreased plasma retinol levels observed in human alcoholics, this may be because of the adequate nutrition provided in the experimental setting or the absence of severe hepatic disease induced by these models. 

**Table 1:** Summary of rodent studies exploring the effect of chronic alcohol consumption on hepatic retinoid status.

Lead author (year of publication) [reference number]	Species (strain)	Hepatic retinoid status
Sato (1981) [18]	Rat (Sprague-Dawley)	Decreased
Leo (1986) [19]	Rat (Sprague-Dawley)	Decreased
Mobarhan (1986) [20]	Rat (Sprague-Dawley)	Decreased
Mobarhan (1991) [21]	Rat (Fischer 344)	Decreased ^#^
Chapman (1992) [22]	Rat (Sprague-Dawley)	Decreased
Wang (1998) [23]	Rat (Sprague-Dawley)	Decreased
Chung (2001) [24]	Rat (Sprague-Dawley)	Decreased
Liu (2002) [25]	Rat (Sprague-Dawley)	Decreased
Chung (2009) [26]	Rat (Sprague-Dawley)	Decreased
Kane (2010) [27]	Mouse (C57BL/6)	Decreased
Luvizotto (2010) [28]	Rat (Fisher 344)	Decreased

^#^ Mobarhan *et al*. (1991) studied old and young rats, and only observed a decreased in hepatic retinoid content in younger animals [21].

In addition to measures of total hepatic retinoid content, the effect of alcohol consumption on individual cell types within the liver has been studied. Perhaps the most significant cell type to consider here is the HSC. This cell type accounts for approximately 5 to 8% of liver cells, yet it stores greater than 90% of hepatic retinoid content (in the form of retinyl ester) within specialized lipid droplets [29]. Rasmussen *et al*. (1985) measured the retinoid content of parenchymal (hepatocyte) preparations (approximately 95% pure) and nonparenchymal cell preparations, reporting a significantly decreased total retinoid content in both cell populations isolated from alcohol-fed rats; however, the largest quantitative drop was observed in the nonparenchymal cell population which contained approximately 10% HSCs [30]. Using a more sophisticated approach to isolate the individual cell types of the rat liver, Cottalasso *et al*. (2003) also reported decreased retinol content associated with alcohol-feeding in hepatocytes, HSCs, Kupffer cells and endothelial cells [31]. In agreement with these animal studies, it has been shown that depletion of hepatic retinoid stores in alcoholics occurs in hepatocytes and HSCs [17]. Taken together, these studies indicate that chronic alcohol consumption is associated with decreased retinoid content in all of the main cell types of the liver, though quantitatively this decrease is largest in HSCs. 

Despite the evidence of depleted hepatic retinoid content in chronic alcoholics, it remains to be established whether dietary vitamin A supplementation would have a beneficial effect on liver pathology. Conflicting results have been published on the effect of vitamin A supplementation to rats chronically consuming alcohol, with one group reporting an enhancement of liver injury (increased lipid accumulation and increased indicators of steatohepatitis) [32,33], and a second group reporting no effect of vitamin A supplementation [34,35]. These opposite results are difficult to reconcile, though differences in study design make direct comparisons problematic. For example, while both studies used the same high amount of vitamin A and dietary alcohol content, the studies were conducted in different strains of rat and over different time periods. This issue is further complicated by reports demonstrating that supplementation with retinoic acid in rats chronically consuming alcohol is associated with decreased liver injury [24,36,37], whereas supplementation with β-carotene exacerbates liver injury [38,39]. Thus, there is conflicting data on the effect of exogenous retinoid supplementation in rats chronically consuming alcohol. The effect of dietary retinoid intake in alcoholic liver disease, be it positive or negative, remains to be resolved. 

In addition to the profound effect that chronic alcohol consumption has on hepatic retinoid levels, it also became apparent that alcohol impacted retinoid levels in extra-hepatic tissues. Interestingly, whereas alcohol consumption was shown to decrease hepatic retinoid levels, these levels are actually increased in extra-hepatic tissues, leading to the concept that alcohol stimulates the mobilization of hepatic retinoid stores to extra-hepatic tissues. To summarize the multiple studies which have investigated this phenomenon, there is evidence to suggest the chronic alcohol consumption increases tissues retinoid levels in specific brain regions, the colon, esophagus, kidney, lung, testes and trachea [18,19,20,21,22,27,40]. It is important to note that although all of the studies mentioned above were focused on the effects of chronic alcohol consumption, it is also known that acute ethanol exposure can affect tissue retinoid levels. Similar to the effects of chronic alcohol consumption, acute alcohol exposure has been shown to precipitate a decline in hepatic retinoid content, with a concomitant increase in extra-hepatic tissue retinoid levels (specifically serum, adipose, and kidney) [27,41,42]. 

As mentioned above, retinoic acid is the active metabolite of dietary vitamin A, which functions as a ligand for nuclear transcription factors to control the expression of more than 500 genes [43]. While the studies discussed thus far indicate that chronic alcohol consumption depletes the liver of its retinyl ester and retinol content, more recent work has also evaluated alcohol’s effect on retinoic acid levels, as reviewed by Napoli [44]. In brief, several studies have described decreased hepatic retinoic acid levels in alcohol-fed rats and mice, as measured by high-pressure liquid chromatography, as well as decreased plasma levels of retinoic acid [23,24,25,26,28,45,46]. Furthermore, data from PAV-1 cells (an immortalized HSC line,) supports the notion that alcohol inhibits retinoic acid formation, as reflected by alcohol’s inhibitory effect on the induction of a retinoic acid-inducible promoter, stimulated upon retinol administration [47]. In contrast, it was recently reported that retinoic acid levels were unchanged in the livers of mice chronically fed alcohol and that extra-hepatic levels (including plasma) were increased, as measured by liquid chromatography-tandem mass spectrometry [27]. Some reviewers have favored this most recent dataset given the more sensitive methodology used [48]; however, it is our opinion that to disregard the previous studies may be premature. Given the importance of retinoic acid in controlling gene expression, and the fact that steady-state levels of retinoic acid may not directly reflect responses in gene expression, it will be important to clarify the effect alcohol has on hepatic and extra-hepatic retinoic acid levels in order to better understand the importance of alcohol-induced changes in retinoic acid as a mechanism of alcohol toxicity, not only in the liver, but also in extra-hepatic tissues.

A further aspect of alcohol’s interaction with vitamin A homeostasis that has not been discussed thus far, is the metabolism of the dietary provitamin A carotenoid, β-carotene; metabolism of which can yield bioavailable vitamin A to meet the body’s needs. An in depth discussion of alcohol’s interaction with metabolism of this nutrient can be found in the 1999 article by Leo and Lieber [49]. In brief, while studies in humans have reported a decrease in plasma β-carotene, detailed analysis and control for nutrient intake suggest there may actually be a relative increase in its circulating levels [49,50,51,52,53,54]; furthermore experimental studies in alcohol-fed baboons have reported a significant increase in circulating and hepatic levels of β-carotene [38]. While it was speculated that the increased levels of β-carotene observed with chronic alcohol consumption might be associated with inhibition of its cleavage into retinal [49], more recent studies have shown that alcohol increases hepatic expression of carotenoid cleavage enzymes (CMO1 and CMO2) which catalyze this reaction (though β-carotene levels were not reported) [28]. As such, increased carotenoid cleavage enzyme expression seems to argue against a mechanism whereby alcohol inhibits cleavage of β-carotene, leading to its accumulation in the liver. Thus it appears that further studies will be required to better understand the unique interaction that alcohol has with β-carotene metabolism, as well as its impact on hepatic retinoid metabolism as a whole. An important caveat should also be added here, specifically, it should be noted that experimental rodents typically have low amounts of β-carotene in their diets, and they are also inefficient at absorbing it, thus extra care should be taken when interpreting and conducting studies on alcohol-fed rodents concerning β-carotene metabolism.

**Figure 2:**
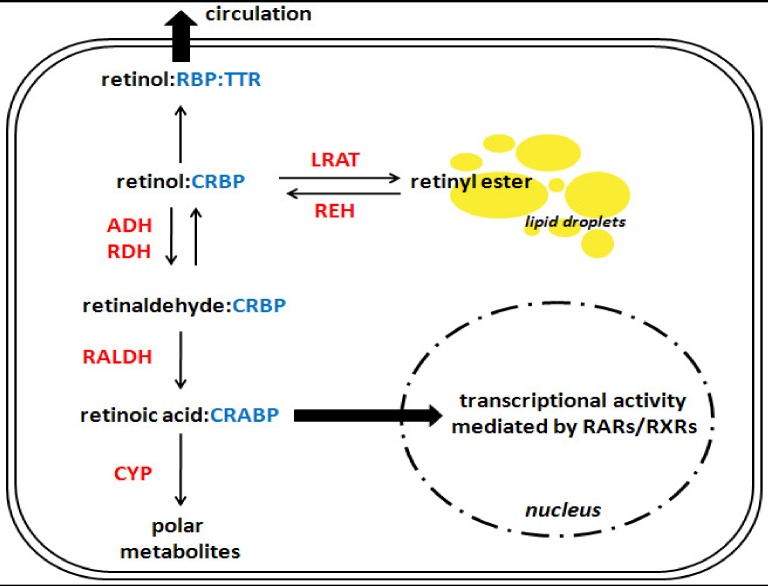
A simplified overview of retinoid metabolism in a hypothetical cell. This scheme reflects retinoid metabolism in a hypothetical cell. The reader should note that these processes do not typically occur within all cells *in vivo*, but have been grouped here for simplicity. Similarly, multiple isoforms exist for many of the binding proteins and enzymes presented below; for a complete review of retinoid metabolism, please refer to the recent review by D’Ambrosio *et al*. [55]. In the cytoplasm, retinol is bound to a cellular retinol-binding protein (CRBP), from this point there are three possible pathways for retinol to take. First, retinol can be transferred to retinol-binding protein (RBP), which itself is bound to transthyretin (TTR), and secreted into the circulation. Second, it can be esterified into retinyl ester by lecithin:retinol acyltransferase (LRAT), and stored in cytoplasmic lipid droplets. Third, it can be metabolized into retinaldehyde and subsequently converted into retinoic acid. Retinoic acid may be bound by a cellular retinoic acid binding protein (CRABP), which can direct it toward the nucleus where it can signal through the nuclear transcription factors retinoic acid receptor (RAR) and retinoid X receptor (RXR), or it can be directed toward catabolism into polar metabolites by various members of the cytochrome P450 family (CYP). Catalytic enzymes are shown in red text; binding proteins are in blue text. ADH: alcohol dehydrogenase; RALDH: retinaldehyde dehydrogenase; RDH: retinol dehydrogenase; REH: retinyl ester hydrolase.

## 4. Interactions between Alcohol and Hepatic Retinoid Metabolism

The studies discussed above provide compelling evidence that chronic alcohol consumption leads to a depletion of hepatic retinoid stores; however, the mechanism to explain this phenomenon remains elusive. Early nutritional studies established that the drop in hepatic retinoid content occurred independently of dietary vitamin A intake, and that malabsorption from the intestine was not responsible. Two hypotheses to explain alcohol’s effect subsequently emerged; (1) that alcohol stimulates mobilization of hepatic retinoid stores to extra-hepatic tissues; and (2) that alcohol stimulates retinoid catabolism [18]. Other posited explanations include reduced uptake of vitamin A into the liver from chylomicron remnants, reduced transfer of retinol from hepatocytes to HSCs, and reduced storage capacity of HSCs [30]. The next section of this review contains a detailed dissection of alcohol’s interaction with the retinoid metabolic pathway, providing a biochemical basis for understanding alcohol’s effect on tissue retinoid levels; a general overview of retinoid metabolism is provided in Figure 2 for the reader’s orientation.

### 4.1. Synthesis and Hydrolysis of Hepatic Retinyl Ester Stores

Vitamin A is stored in HSCs in the form of retinyl ester, which is packaged into cytoplasmic lipid droplets. In times of dietary vitamin A insufficiency, stored retinyl ester can be hydrolyzed into retinol, which is transferred to hepatocytes, packaged with RBP, and secreted into the circulation in order to meet the body’s need for vitamin A. Hydrolysis of retinyl ester is catalyzed by a retinyl ester hydrolase (REH); however, the exact molecular identity of this enzyme activity in HSCs remains unclear [29]. The effect of ethanol on REH activity has been studied in vitro using liver homogenates, revealing that alcohol exerts a dose dependent increase in REH activity but acetaldehyde can inhibit REH [56]. These opposing results are difficult to interpret in the context of alcohol consuming mice, which would be expected to have elevated concentrations of alcohol and its metabolite, acetaldehyde. Perhaps data that is more representative of alcohol’s effect on REH activity in vivo comes from studies in rats chronically fed alcohol; in this case, hepatic microsomes isolated from alcohol-fed animals had significantly decreased rates of REH activity, as compared to controls [21]. This data suggests that mobilization of hepatic retinyl ester is unlikely to be mediated by a direct effect of alcohol on REH activity; however, an indirect mechanism has been proposed. In this case, it has been hypothesized that alcohol increases hepatic levels of apo-CRBPI (cellular retinol binding protein, type 1), a high concentration of which relative to holo-CRBPI, is proposed to stimulate hydrolysis of retinyl ester and inhibit its esterification [57,58]. Though definitive data is lacking, this hypothesis is supported by evidence indicating that hepatic CRBPI mRNA expression is upregulated by ethanol exposure, and that CRBPI-null mutant mice are resistant to alcohol-induced depletion of hepatic retinoid stores [25,27]. As indicated above, it is also possible that alcohol has an effect on the synthesis of retinyl ester within HSCs, though it has been shown that alcohol has no effect on LRAT (lecithin:retinol acyltransferase) mRNA expression and that synthesis of retinyl palmitate from retinol is unaffected by alcohol feeding [25]. Take together, it would appear that there is no definitive evidence to conclude that alcohol has a direct effect on the rates of hydrolysis or synthesis of retinyl ester in the liver, and that depletion of hepatic retinyl esters stores is secondary to alcohol’s effect upon another step in the retinoid metabolic pathway.

### 4.2. Synthesis of Retinoic Acid

As shown in Figure 2, retinoic acid is synthesized from retinol in a two-step process in which retinol is oxidized to retinaldehyde followed by the subsequent oxidation of retinaldehyde to retinoic acid. The synthesis of retinaldehyde from retinol can be catalyzed by several enzymes, which can be broadly split into two categories: (1) medium-chain alcohol dehydrogenases with specific activity for retinol (e.g., ADH1, ADH3, ADH4); and (2) retinol dehydrogenases which are members of the short-chain dehydrogenase/reductase family (e.g., RDH1 and RDH10) [59]. It is currently thought that the retinol dehydrogenase enzymes function in hepatic retinol metabolism at physiological levels, and that alcohol dehydrogenases produce retinoic acid only when excess retinol levels are present [60]. In the early literature, the role of alcohol inhibiting the conversion of retinol to retinaldehyde by ADH1 was focused on because of ADH1’s high expression level in the liver and its important role in ethanol metabolism. Interestingly, *in vitro* data indicated that ethanol is a potent inhibitor of alcohol dehydrogenase catalyzed oxidation of free retinol to retinaldehyde, particularly ADH1 [61,62]; however this effect is apparently attenuated when retinol is bound to CRBP [63,64]. Thus, if we accept that the majority of cellular retinol is bound by CRBP, it would appear that retinol metabolism by ADH1 is not an important pathway in the alcoholic liver. This conclusion is supported by recent data which shows that acute inhibition of ADH with 4-methylpyrazole did not change alcohol’s effect on cellular retinoic acid levels [27]. Interestingly though, the effect of alcohol on cellular retinoic acid concentration was blocked by inhibition of retinol dehydrogenase, suggesting that alcohol may affect this pathway [27].

The second step in the synthesis of retinoic acid from retinol is the oxidation of retinaldehyde to retinoic acid (Figure 2); this reaction is catalyzed by members of the retinaldehyde dehydrogenase family (RALDH1 [ALDH1a1], RALDH2 [ALDH1a2] and RALDH3 [ALDH1a3]) [59]. Though this step has been less studied with regard to alcohol, it has been reported that hepatic microsomes isolated from alcohol-fed rats have increased retinaldehyde dehydrogenase activity, which was associated with the depletion of hepatic retinoid observed in these animals [65].

In summary, when considering the synthesis of retinoic acid one must keep in mind that it is a two-step process, in which the first step uses retinol as a substrate and is potentially affected by alcohol, whereas the second step utilizes retinaldehyde as a substrate and is potentially affected by acetaldehyde. However, there is currently no definitive experimental evidence to support the concept that alcohol may inhibit retinoic acid synthesis by inhibiting its oxidation from retinol to retinaldehyde. Indeed, while the idea exists that oxidation of retinol is the rate-limiting step in retinoic acid synthesis, and is thus essential in controlling cellular levels of retinoic acid, a different regulatory mechanism has been proposed. In this model, retinoic acid levels are controlled by two factors: substrate availability and catabolism. Specifically, Ross and colleagues have proposed that levels of the retinoic acid precursor molecule, retinol, are tightly controlled by its sequestration into retinyl ester, catalyzed by LRAT, and secondly, retinoic acid itself is regulated by its catabolism, mediated by cytochrome P450 enzymes [66]. The effect of chronic alcohol consumption on the catabolism of retinoic acid is discussed in the next section.

### 4.3. Catabolism of Retinoic Acid

Under physiological conditions, the catabolism of retinoic acid is catalyzed by cytochrome P450 enzymes. While the actions of CYP26A1, 26B1 and 26C1 in retinoic acid catabolism have been well established, particularly during embryogenesis, several other cytochrome P450 enzymes have also been posited to metabolize retinoic acid, including several members of the CYP2C family [67]. In the context of chronic alcohol consumption, decreased levels of hepatic retinoic acid have often been associated with enhanced breakdown of this transcriptionally active vitamin A metabolite. There are three lines of evidence which support this notion, (1) decreased steady state levels of retinoic acid in the liver of alcohol-fed rodents (see above); (2) direct evidence of increased retinoic acid breakdown [27,45,46]; and (3) increased levels of polar retinoic acid metabolites, such as 4-oxo-retinoic acid and 18-hydroxy-retinoic acid, in the liver of alcohol-fed rodents [25,26,45,68]. Chronic alcohol consumption is known to induce the cytochrome P450 enzyme CYP2E1 [69]. Enhanced cytochrome P450 activity was associated with decreased hepatic retinoid content since some of the first feeding experiments in rodents were performed [18], the hypothesis that retinoic acid is metabolized in the alcoholic liver by CYP2E1 has been borne out by studies using a CYP2E1 inhibitor (chlormethiazole; CMZ). Treatment with this compound has been shown to normalize hepatic retinoic acid levels and prevent the appearance of retinoic acid metabolites in a dose-dependent manner [25,26,68]. Interestingly, not only did CMZ treatment prevent alcohol-induced decreases in hepatic retinoic acid levels, but it also normalized hepatic retinol and retinyl ester levels [25]. This intriguing result suggests that by preventing the breakdown of retinoic acid within the liver, alcohol-induced depletion of retinol and retinoic acid can also be avoided, thus the increased metabolism of retinoic acid within the alcohol-exposed liver could be the main driving force behind the depleted level of hepatic retinoids, such that retinyl ester stores are channeled through retinol oxidation to retinoic acid which is subsequently broken down by CYP2E1. 

Our goal in this section was to summarize the numerous studies which have sought to explain the mechanism through which alcohol consumption depletes hepatic retinoid levels. Other authors have attempted to synthesize the pleiotropic effects of alcohol into one scheme, and here we defer to them [27,70]. Suffice to say, *in vitro* studies provide compelling evidence that alcohol can disrupt several steps in the metabolism of retinol; however, the effect *in vivo* is less clear. It is likely that depletion of hepatic retinoid stores is a dynamic process and that mobilization to extra-hepatic tissues and enhanced retinoid catabolism both contribute to this effect. An important issue to be resolved in this regard is the relative contribution of these mechanisms, both in terms of time and quantity. A further important issue is the effect that alcohol-associated alterations in retinoid homeostasis have on alcohol-induced disease development; this is the subject of the next section.

## 5. Effect of Altered Retinoid Homeostasis on Alcohol-Induced Disease

One of the organs most affected by chronic alcohol consumption is the liver; the progression of disease in chronic alcoholics typically begins with fatty liver, which can then develop into steatohepatitis, fibrosis, and cirrhosis. The development of fibrosis in liver disease involves the activation of HSCs, which transition from quiescent vitamin A-storing cells to myofibroblasts [71]. Significantly, a prerequisite step in the activation of HSCs is the loss of their retinyl ester stores [29], thus at a superficial level we can draw an association between alcohol-induced depletion of hepatic retinoid stores and the progression of alcohol-induced disease; however, the cause and effect relationship between alcohol-induced depletion of hepatic retinoid and development of disease has not been definitively ascertained. Two leading hypotheses have been forwarded which explore this relationship, one we call here the retinoid insufficiency hypothesis and the second, the toxic burst hypothesis [25]. In the retinoid insufficiency hypothesis, it is postulated that alcohol-induced loss of hepatic retinoid content results in a state of hepatic retinoid insufficiency, thus disrupting essential retinoid-dependent cellular functions and precipitating disease. In this context, hepatic retinoid stores can be viewed as being protective against disease development. In the toxic burst hypothesis, alcohol-induced aberrations in hepatic retinoid metabolism results in a toxic burst of transcriptionally active retinoid metabolites, presumable generated by CYP2E1, which directly contribute to the development of alcoholic liver disease [25]. 

As stated above, these hypotheses have been largely untested, though data from the existing literature can be found to support each of them. For example, the retinoid insufficiency hypothesis is supported by several studies showing that interventions that ameliorate the development of alcohol-induced disease are associated with the preservation of hepatic retinoid levels [68,72,73]. With regard to the toxic burst hypothesis, Dan *et al*. studied the toxicity of polar retinol metabolites in the pathogenesis of alcohol-induced liver disease, finding them to be cytotoxic to HepG2 cells and primary rat hepatocytes [74]. It is also known that blocking CYP2E1, which inhibits the formation of retinoic acid metabolites, protects against the development of alcoholic liver disease in animal models [75,76], though it is important to remember that CYP2E1 has an important role in alcohol metabolism and its inhibition may have other effects beyond normalizing retinoid metabolism. While these studies can be interpreted to favor a specific hypothesis, the reader is reminded that these associations are largely correlative and definitive data is lacking.

A further complication of chronic alcoholism is the development of hepatocellular carcinoma (HCC); analysis of retinoid content in HCC tissue and surrounding noncancerous tissue from patients with a history of alcoholism revealed a low retinoid content, which was more pronounced in HCC tissue, leading the authors to conclude that depleted hepatic retinoid content may be a factor in HCC development in alcoholics [17]. It is not only an increased risk of liver cancer that has been associated with alcoholism; alcohol consumption is also associated with increased rates of several types of cancer in addition to HCC, including cancer of the upper aerodigestive tract, colorectal cancer and female breast cancer [77]. Furthermore, some of these cancers have been linked with changes in retinoid homeostasis, such as tracheal squamous metaplasia [78,79,80]. The reader is directed towards the review by Wang, which specifically address the associations between alcohol, cancer and vitamin A [70]. 

An understudied aspect of alcohol’s effect on retinoid homeostasis, with respect to alcohol-induced disease, is its effect on other organ systems. While most studies have focused on the liver, it is known that chronic alcohol consumption affects retinoid homeostasis in extra-hepatic tissues, generally leading to an increase in retinoid levels (discussed above). For example, an association has been made between male sterility in alcoholics and altered testicular retinoid homeostasis [81,82]. As such, examination of altered retinoid signaling in extra-hepatic tissues of chronic alcoholics may be of interest for understanding the pathology of alcohol-induced diseases in addition to that of the liver.

## 6. Summary

Alcohol consumption has a profound effect on whole body retinoid homeostasis. This has been most carefully studied in the liver, where chronic alcohol consumption is associated with a depletion of hepatic retinyl ester and retinol levels. Though there is compelling data to support this observation in humans and rodent models, the mechanisms which underlie alcohol’s effect have not been definitively established; indeed, it appears that alcohol consumption has pleiotropic effects on hepatic retinoid metabolism. It is also important to emphasize that the effects of chronic alcohol consumption are tissue specific, such that alcohol is associated with a decrease in hepatic retinoid content, yet extra-hepatic retinoid levels are elevated. While future studies should yield an improved mechanistic understanding of the interactions between alcohol and retinoid metabolism, what will be most significant is definitive studies aimed at establishing if alcohol’s effect on hepatic retinoid homeostasis is directly linked with the development of alcoholic liver disease. The growing trend towards alcohol feeding studies in genetically-engineered mutant mice should aid the discovery process, as well as the use of sensitive analytic methodologies for retinoid acid measurement.
